# COVID-19 reinfections among naturally infected and vaccinated individuals

**DOI:** 10.1038/s41598-022-05325-5

**Published:** 2022-01-26

**Authors:** Sezanur Rahman, M. Mahfuzur Rahman, Mojnu Miah, Mst Noorjahan Begum, Monira Sarmin, Mustafa Mahfuz, Mohammad Enayet Hossain, Mohammed Ziaur Rahman, Mohammod Jobayer Chisti, Tahmeed Ahmed, Shams El Arifeen, Mustafizur Rahman

**Affiliations:** 1 Virology Laboratory, Infectious Diseases Division, icddr,b: International Centre for Diarrhoeal Disease Research, Bangladesh, 68 Shaheed Tajuddin Ahmed Sarani, Mohakhali, Dhaka, 1212 Bangladesh; 2 Nutrition and Clinical Services Division, icddr,b: International Centre for Diarrhoeal Disease Research, Bangladesh, Mohakhali, Dhaka, 1212 Bangladesh; 3 Maternal and Child Health Division, icddr,b: International Centre for Diarrhoeal Disease Research, Bangladesh, Mohakhali, Dhaka, 1212 Bangladesh

**Keywords:** SARS-CoV-2, Viral infection, Epidemiology

## Abstract

The protection against emerging SARS-CoV-2 variants by pre-existing antibodies elicited due to the current vaccination or natural infection is a global concern. We aimed to investigate the rate of SARS-CoV-2 infection and its clinical features among infection-naïve, infected, vaccinated, and post-infection-vaccinated individuals. A cohort was designed among icddr,b staff registered for COVID-19 testing by real-time reverse transcriptase-polymerase chain reaction (rRT-PCR). Reinfection cases were confirmed by whole-genome sequencing. From 19 March 2020 to 31 March 2021, 1644 (mean age, 38.4 years and 57% male) participants were enrolled; where 1080 (65.7%) were tested negative and added to the negative cohort. The positive cohort included 750 positive patients (564 from baseline and 186 from negative cohort follow-up), of whom 27.6% were hospitalized and 2.5% died. Among hospitalized patients, 45.9% had severe to critical disease and 42.5% required oxygen support. Hypertension and diabetes mellitus were found significantly higher among the hospitalised patients compared to out-patients; risk ratio 1.3 and 1.6 respectively. The risk of infection among positive cohort was 80.2% lower than negative cohort (95% CI 72.6–85.7%; p < 0.001). Genome sequences showed that genetically distinct SARS-CoV-2 strains were responsible for reinfections. Naturally infected populations were less likely to be reinfected by SARS-CoV-2 than the infection-naïve and vaccinated individuals. Although, reinfected individuals did not suffer severe disease, a remarkable proportion of naturally infected or vaccinated individuals were (re)-infected by the emerging variants.

## Introduction

Bangladesh observed the third wave of COVID-19 pandemic and faced a record upsurge during June–September 2021, fueled by the highly contagious Delta variant^1^. Many of these COVID-19 positive cases had reported previous experience with natural infection or vaccination. In Bangladesh, the COVID-19 vaccination started on 27 January 2021 with COVISHIELD™ (ChAdOx1 nCoV-19 Corona Virus Vaccines manufactured by Serum Institute of India Pvt Ltd). Until July 2021, six additional COVID-19 vaccines (mRNA-1273, BNT162b2, Sputnik V, Ad26.COV2.S, BBIBP-CorV/Vero Cells, and CoronaVac) have been approved by the Govt. of Bangladesh^2^. Recent studies suggest that natural infections are protective against reinfection at least for 8–12 months^3^ and vaccination confers strong resistance against variants of concern, including the Delta variant. However, even with high vaccine coverage, many countries face multiple waves with faster high altitude spread than the previous^1,4–7^. Therefore, the protection against the new variants with pre-existing antibodies due to natural infection or vaccination becomes a global concern^8–10^.

More than 95% of symptomatic cases develop antibodies within 14 days, and by day 30, 100% symptomatic and 45% asymptomatic cases become fully seroconverted^11^. But, the concentration of neutralising antibodies is another factor to confer protection against SARS-CoV-2 reinfection^12^. Recently, the number of reinfection cases have been increasing globally^13^. Although, CDC considered symptomatic infection < 35 Ct-value with ≥ 45 days interval between two rRT-PCR tests as reinfection cases^14^; Tang et al. identified reinfection cases within 19 days by different PANGO Lineage SARS-CoV-2^15^. Therefore, how long an individual is protected from further SARS-CoV-2 infection after recovering from COVID-19, becomes an important research question during this prolonged pandemic.

Recent studies showed that complete vaccination was effective against SARS-CoV-2 even for emerging variants; and infection was significantly lower among vaccinated individuals than non-vaccinated^16–19^. In contrast, several studies showed low vaccine efficacy against Delta (B.1.617.2) variant compared to Wuhan (B.1) or Alpha (B.1.1.7) variants^20–22^. Another study from a town in Massachusetts showed that among 469 cases, mostly infected by the Delta variant, 74% were fully vaccinated^23^. While, another study identified a small fraction of vaccine breakthrough infections in USA^24^; in contrast, data from densely populated and low vaccine coverage regions is limited^25^. Therefore, it is important to evaluate the vaccine's effectiveness against emerging variants.

Although host immunity^26,27^ and chance of exposure^28^ are the main factors, genomic evidence shows that recurrent cases were infected with phylogenetically distinct SARS-CoV-2 strains^29–36^. Antibodies against the spike protein were effective in inhibiting SARS-CoV entry into the host cell; however, mutations in the receptor-binding domain of S-protein helps them to escape host immunity and lead to the emergence of new variants^37–39^. Emerging new variants are not only capable of escaping immunity and causing reinfection but also show increased transmissibility, severity and mortality^40–42^. Therefore, molecular surveillance for variant monitoring is crucial, and several countries and research organisations have already started surveillance programs^1,43^.

icddr,b (International Centre for Diarrhoeal Disease Research, Bangladesh) is an international health research institution with approximately 4000 national (~ 95%) and international clinicians, health workers, scientists and non-scientific staff. This organisation has been providing an extensive staff-clinic facility for staff and their family members. Since March 2020, icddr,b staff-clinic started COVID-19 testing and treatment support. Taking advantage of this ongoing COVID-19 testing and treatment facility, we designed a cohort study and investigated the frequency of SARS-CoV-2 infection among infection naïve, previously infected, vaccinated, vaccinated with previous infection and non-vaccinated staff. Here we provide comparative data analysis of (1) SARS-CoV-2 infections between COVID-19 negative and positive cohort; (2) clinical features and genomic variations between first episode and reinfection; and finally (3) data on SARS-CoV-2 infection among vaccinated and unvaccinated persons.

## Results

**Figure 1:**
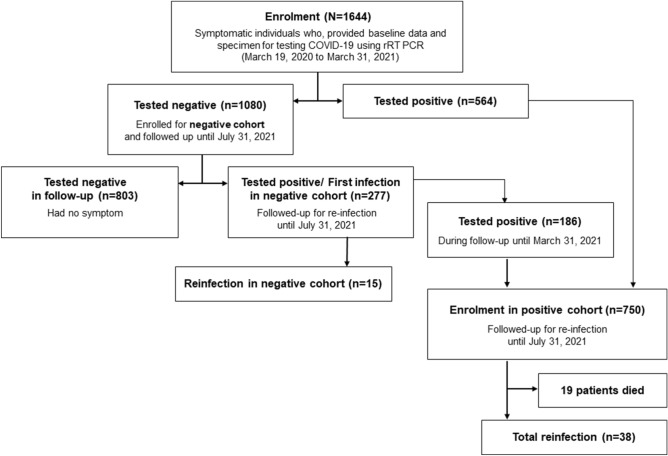
Study profile (participants were enrolled until 31 March 2021, and all participants were followed up until 10 July 2021).

From 19 March 2020 to 31 March 2021, a total of 1644 staff of icddr,b were enrolled for the COVID-19 testing cohort (Fig. 1). Males were predominant (n = 939; 57.2%), and the mean age was 38.4 years (median 37 years) (Table 1). 1080 (65.7%) participants were tested negative using rRT-PCR for SARS-CoV-2 and enrolled as a negative cohort. The positive cohort includes 750 positive cases, 564 from baseline and 186 from the negative cohort (Fig. 1).

**Table 1:** Characteristics of study participants by baseline cohort allocation and follow-up.

	All participant (N = 1644)	Baseline		Follow-up	
		Negative cohort (N = 1080)	Positive cohort (N = 750)	Negative cohort^a^ (N = 277)	Positive cohort^b^ (N = 38)
**Gender**					
Male	940 (57.2%)	609 (56.4%)	446 (59.4%)	167 (60.3%)	27 (71.1%)
Female	704 (42.8%)	471 (43.6%)	304 (40.6%)	110 (39.7%)	11 (28.9%)
**Age (year)**					
Mean	38.4	38.3	39.1	40.5	40.2
Median (Std. deviation)	37.0 (10.2)	37.0 (10.4)	38.0 (9.9)	40.0 (9.9)	38.0 (10.7)
IQR	30.0–45.8	30.0–45.0	31.0–46.7	32.0–48.0	30.7–50.3
**Cause for testing**					
Influenza like illness		587 (54.4%)	607 (80.8%)	203 (73.3%)	32 (84.2%)
Contact with COVID-19 case		112 (10.4%)	79 (10.7%)	38 (13.7%)	6 (15.8%)
Asymptomatic periodic test		381 (35.2%)	64 (8.5%)	36 (13.0%)	–
**Symptoms**^**c**^					
Fever		358 (33.1%)	466 (62.1%)	180 (64.9%)	29 (76.3%)
Cough		383 (35.4%)	328 (43.7%)	130 (46.9%)	21 (55.3%)
Short of breath		44 (4.1%)	25 (3.3%)	13 (4.7%)	2 (5.3%)
Sore throat		77 (7.1%)	39 (5.2%)	11 (4.0%)	3 (7.9%)
Other symptom		44 (4.1%)	49 (6.5%)	22 (7.9%)	5 (13.2%)
**Day intervals**^**d**^					
Mean (Std. error of mean)				173.6 (7.7)	195.6 (19.4)
Median (Std. deviation)				138.0 (128.0)	156.5 (119.5)
IQR				68–257	93.5–287.8
Minimum				1	47
Maximum				566	460

^a^First infections.

^b^Reinfections.

^c^Symptomatic case.

^d^During follow-up.

### Negative cohort

In the negative cohort baseline, 609 (56.4%) participants were male, and the mean age of the participants was 38.3 years (Table 1). As far as causes of testing were concerned, 112 (10.4%) participants were tested for having confirmed contact with other COVID-19 patients, while 586 (54.3%) participants were tested for having influenza-like illness and 382 (35.4%) for asymptomatic routine check-up. Cough (n = 383, 35.4%), fever 358 (33.1%), sore throat 77 (7.1%) and shortness of breath 44 (4.1%) were common symptoms. Other symptoms including headache, vomiting, diarrhoea, and asthma were also recorded.

**Figure 2:**
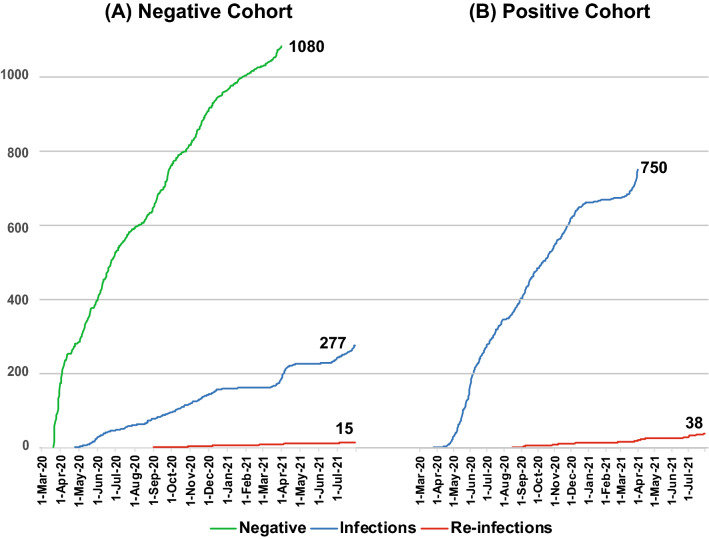
Cumulative number of cases in Y-axis from (**A**) negative cohort and (**B**) positive cohort and X-axis indicate timeline (date). The green line indicates a cumulative number of negative cohort enrolment; the blue line indicates a cumulative incidence of infection (first infection in negative cohort and enrollment in the positive cohort), and the red line indicates a cumulative number of reinfection.

During follow-up till 31 July 2021 (0.99-person year), 277 cases (25.8 infections per 100 person-year) were infected with SARS-CoV-2 (Fig. 2A), where the male (n = 167; 60.3%) were predominant. Both mean (40.5 yeas) and median (40.0 years) age for infected cases were slightly higher than baseline participants. Also, the symptomatic follow-up cases were more likely to be positive (n = 203, 73.3%) than asymptomatic cases (n = 36). Among infected participants, 180 (64.9%) came with fever, while 130 (46.9%) had a cough. The mean interval between negative cohort enrolment and infection was 173.6 days (median, 138.0).

### Positive cohort

A total of 750 SARS-CoV-2 positive cases were followed up under the positive cohort for reinfection identification (Fig. 2B). Gender (n = 446, 59.5% male) and age distribution (mean 39.1 years and median 38.0 years) for positive cohort participants were similar to the negative cohort (Table 1). The symptomatic cases were much highly represented (n = 607, 80.9%) in the positive cohort as opposed to the negative cohort (n = 586, 54.3%). The symptomatic cases in the positive cohort were more likely to present with fever (n = 466, 62.1%) and cough (n = 328, 43.7%) than the other symptoms. The mean Ct-value of the RdRp gene for symptomatic cases 22.9 (median 20.4, IQR 17.0–27.6) was significantly (p = 0.01) lower than asymptomatic (mean 25.3, median 22.8, IQR 16.8–35.1).

Among all positive cases, 207 (27.6%) were admitted to the icddr,b COVID-19 management hospital and received treatment (in-patient group) according to the national guideline on clinical management of COVID-19^44^ and 19 of them died. Thus the overall case fatality rate was 2.5% (19/750). Also, 232 (30.9%) positive cases received medication and support from the staff-clinic but were not admitted (out-patient group). For the rest of the cases (n = 312, 41.6%), no medical attention was required due to low-mild symptoms, and they were recovered after homestay.

**Figure 3:**
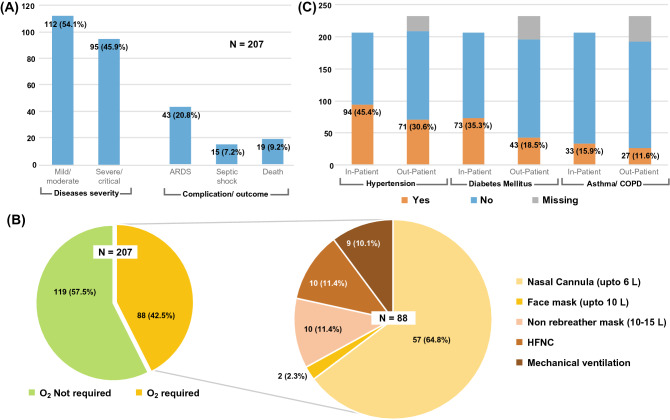
Clinical features of the positive cohort. (**A**) Diseases severity, complication, and outcome of in-patient group. (**B**) Oxygen requirement of in-patient group. (**C**) Comorbidity of COVID-19 positive in-patient (hospitalised, n = 200) and out-patient (not hospitalised but seek treatment in staff clinic, n = 207) group.

Disease severity, complication/outcome was recorded only for the in-patient group (n = 207) (Fig. 3A); where 95 (45.9%) cases had severe disease based on national guideline^44^. Among them, Acute Respiratory Distress Syndrome (ARDS) had developed in 43 (20.8%) and septic shock in 15 (7.2%) participants. 88 (42.5%) participants required oxygen support (Fig. 3B); through a nasal cannula (n = 57, 64.8%), mask (n = 12, 13.6%), through High Flow Nasal Cannula (n = 10, 11.4%) and mechanical ventilation (n = 9, 10.1%).

Comorbidities (hypertension, diabetes mellitus, and asthma/COPD) were recorded for both in-patient and out-patient groups (Fig. 3C). Hypertension (RR 1.4; 95% CI 1.1–1.7) and diabetes mellitus (RR 1.5; 95% CI 1.3–1.8) were found significantly (p ≤ 0.001) higher among the in-patient group than out-patient. Asthma/COPD was also found in a higher number in the in-patient group but was not statistically significant.

### Reinfection

From the positive cohort, 731 (excluding death cases) COVID-19 positive participants (episode-1) were followed up for 0.91-person-years, and 38 (5.7 reinfections per 100 person-year) were identified as reinfection (episode-2) with SARS-CoV-2. The risk of reinfection in the positive cohort was significantly lower (RR 0.3; 95% CI 0.19–0.35; p < 0.001) than primary infection in the negative cohort. The mean Ct-value of RdRp gene in episode-1 of reinfected cases (29.6, median 34.5, IQR 20.4–36.3) was significantly (p < 0.001) higher than non-reinfected cases (22.9, median 20.4, IQR 16.8–28.2). During reinfection, the mean Ct-value of RdRp gene was 26.3 (median 26.2, IQR 18.9–33.3), which was lower than episode-1 but not statistically significant (p = 0.1). Mean intervals for reinfection (episode-1 to episode-2) was 195.6 days, and the median was 156.5. The earliest day of reinfection was 47 days after the primary infection, and the longest was 460 days (Appendix 1E).

Among reinfected cases, 9 (23.7%) received one dose of the COVISHIELD™ vaccine at least 34 days (mean 68.6) before reinfection, while three received full vaccination before reinfection. At least one comorbidity among obesity, diabetes, asthma, heart disease, lung disease, and high blood pressure (BP) was present in 19 (50%) reinfection cases. During episode-2, confirm contact with another COVID-19 patient was higher than episode-1 (14 vs. 8). Although several mild symptoms were recorded for primary infection and reinfection (Appendix 1E), no one required hospitalisation except one (case-25) during primary infection and one (case-16) during reinfection. The hospitalisation case during primary infection (case 25) and reinfection (case 7) was not severe and did not require oxygen support, while one hospitalisation case (case 16) during reinfection was critical and required oxygen support through High-Flow Nasal Cannula (HFNC) having oxygen flow up to 20 L/min in Intentive Care Unit^45^.

**Figure 4:**
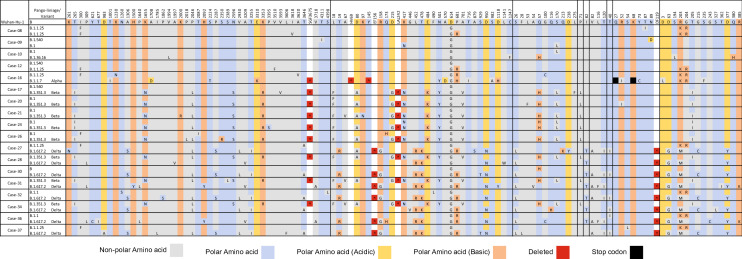
Amino acid substitution map of reinfected cases (for both infection and reinfection events) confirming reinfection by genetically distinct SARS-CoV-2.

Nearly complete genome sequences from both episode-1 and episode-2 infections were retrieved from 18 reinfections out of 38 cases. The amino acid sequence comparison among the strains from episode-1 and episode-2 with reference Wuhan strain (GenBank Accession# NC_045512.2) is shown in Fig. 4. The sequencing results revealed that the reinfection was caused by a different (PANGO Lineage) strain than the first infection, except one (case-08). For case numbers 09, 10 and 12, both episodes were caused by the same Wuhan-like variant while others were caused by different variants than first infections. The case-16 was infected by the Wuhan-like variant in episode-1 and by the Alpha variant in episode-2, which was the only severe case during reinfection. Case numbers 17, 20, 21, 24, 26 were reinfected by Beta variant, and case numbers 27, 30, 32, 36, 37 were reinfected by Delta variant, while all these cases were first infected by Wuhan-like variant. Case numbers 28, 31 and 34 were infected by Beta in episode-1 and reinfected by Delta variant. Case-08 was the only case infected and reinfected by the same PANGO Lineage (B.1.1.25); however, three amino acids were different between episode-1 and episode-2. The case was asymptomatic and tested positive on 6 September 2020 as confirmed contact with another COVID-19 patient. After 20 days, on 26 September, the follow-up rRT-PCR test was negative. On 23 October 2020, after 47 days from episode-1, the case was found positive with high fever, cough, diarrhoea, and muscle aches. Thus, the case was confirmed as reinfection by the definition provided in Appendix 1B.

### SARS-CoV-2 infection among vaccinated individuals

A subset of patients were analyzed to calculate the association between vaccination and (re)-infection. From 1 May 2021 to 31 July 2021, 381 symptomatic staff provided specimens for SARS-CoV-2 rRT-PCR testing; among them, 115 (30.2%) received at least one dose, and 96 (25.2%) received full doses of COVISHIELD™ vaccine before the test date. Among full dose vaccinated individuals, 37 (38.5%) were infected and among non-vaccanated 96 (36.1%) were infected; while the risk ratio between vaccinated and non-vaccinated individuals was statistically insignificant (RR = 1.08, 95% CI 0.76–1.534 and *p* = 0.66). Also risk ratio between full dose vaccinated and single dose vaccinated individuals was statistically insignificant (Appendix-1). Mean intervals from vaccination date (full dose) to SARS-CoV-2 positive test date was 65.1 (median 66.0, IQR: 51.3–82.0) days.

Among the positive cases, no one required medical attention except three who had co-morbidites. Two vaccinated individuals had high blood pressure, attended the hospital and were discharged with conventional COVID-19 medication after a few hours of observation. Another case was non-vaccinated and had asthma with thyroiditis, which required oxygen support with a nasal cannula.

## Discussion

This study represents infection and reinfection events of SARS-CoV-2 by a robust prospective cohort study from a densely populated country, Bangladesh. We did a systematic search to identify studies that described clinical characteristics and/or potential factors and/or rates for recurrence of positive SARS-CoV-2 (Appendix 1A). A total of 22 cohorts were identified, and no cohort confirmed reinfection by whole-genome sequencing. In addition, among 118 published case reports, only 12 confirmed reinfections by whole-genome sequencing. Therefore, our cohort is unique in identifying reinfection cases with genomic evidence, which is essential to confirm true reinfections. Here we have provided evidence that: (1) being naturally infected confers better protection against the SARS-CoV-2; (2) SARS-CoV-2 infection was associated with an 80% lower risk for concurrent infection, with more than six-month protective effect after primary infection; (3) COVISHIELD™ vaccine showed reduced effectiveness against new variants of SARS-CoV-2.

In this cohort, both symptomatic and asymptomatic cases were enrolled for rRT-PCR test; however, symptomatic cases were 1.3 times more likely to be positive than asymptomatic cases. Also, the high representation of symptomatic cases in the positive cohort compared to the negative cohort suggests that in a population and setting that this sample is representative of, SARS-CoV-2 is a more likely etiology for influenza-like illness. The significantly higher infection rate among infection naïve participants than patients previously infected with SARS-CoV-2 indicates possible protection by natural infections. Mean rRT-PCR Ct-value during episode-1 of reinfected cases was significantly higher than others. Also, the fact that the mean Ct-value of the symptomatic cases was found to be significantly lower than asymptomatic ones, similar to another study^46^, indicates that a relatively low Ct-value or high viral load was required to develop COVID-19-like symptoms^47–49^ as well as for seroconversion^11,50^.

Although seroconversion and concurrent protection against any pathogen depend on several host factors, a median protective effect of over 5-months protective effect was observed in this study, confirming previous studies^51–53^. Additionally, previous infection history was associated with an 80% lower risk of further infection; a similar result was observed in previous studies^45,54,55^. However, seroconversion is not the only protective measure because, after a few months, the amount of neutralising antibodies decline^56,57^ while cellular immunity induced by natural infection plays a significant role in preventing subsequent infections^58^.

On the other hand, vaccination after SARS-CoV-2 infection increased T-cell immunity, antibody-secreting memory B-cell response to the spike protein, and neutralising antibodies effectivity even after the first dose of mRNA-based vaccines (Pfizer-BioNTech or Moderna)^59–61^. While our data showed vaccination with COVISHIELD™, a vector based recombinant DNA vaccine was unable to prevent infection or reinfection^62^. Reynolds et al. and Stamatatos et al., provided evidence of cross-variant neutralisation of Alpha (B.1.1.7) and Beta (B.1.351) variants by the Pfizer-BioNTech or Moderna vaccination after natural infection. However, molecular evidence of reinfection from this study, along with others^63^ indicating that genetically distinct strains or new variants can escape immunity whether it was achieved by natural infection or vaccination. Although existing data showed that vaccinated COVID-19 patients are lower at risk for hospitalization^64^, our data failed to correlate vaccination status with hospitalisation and variants due to a low number of vaccinated individuals infected with different variants. Therefore, a large study with vaccinated, non-vaccinated, hospitalised, and non-hospitalised COVID-19 patent infected with different SARS-CoV-2 variants is needed.

In Bangladesh, the first COVID-19 case was identified on 8 March 2020, and since then icddr,b started SARS-CoV-2 rRT-PCR test for staff with influenza-like illnesses. Upon the first case identification among icddr,b staff on 19 March 2020, the cohort was started. Therefore, an antibody test against SARS-CoV-2 was not required to confirm infection naïve participants. On the other hand, rRT-PCR test is considered to be the gold standard for its high sensitivity and specificity, which was used to define the cohort (negative or positive). Hence there was an inconsiderable chance for selection bias in this study. But there might be some Hawthorne effect in the patients’ self reporting of symptoms and cause of testing due to the limitations imposed by the phone-call registration system for COVID-19 testing. Also, reinfected cases were extensively interviewed after the second infection, allowing the possibility of recall bias. Despite the minimal Hawthorne effect and recall bias, this was a robust cohort study from a densely populated country like Bangladesh in order to understand SARS-CoV-2 infection dynamics.

In summary, our data indicate that prior infection ensures some degree of protection against SARS-CoV-2 reinfections. However, emerging variants could (re)-infect naturally infected or vaccinated individuals. Therefore, along with vaccination, other non-pharmaceutical interventions and protective measures need to be implemented for infection control. Our data also warrant evaluation of the vaccine effectiveness against emerging variants.

## Methods

### Specimen and data collection

This prospective cohort study was designed among icddr,b staff registered for COVID-19 testing. During registration over phone call, the staff-clinic received clinical and behavioural data from the participants. These data include cause of testing, age, sex, symptom, date of symptom onset, travel history, and possible contact history with confirmed COVID-19 patients. In terms of the cause of testing all participants were classified into; (1) contact cases (having symptoms or not), (2) symptomatic/influenza-like illness (do not have contact history), and (3) asymptomatic (for routine check-up). Since the national COVID-19 vaccination started, the staff-clinic included and updated the vaccination statuses of the staff in the database. After registration, all suspected cases provided nasopharyngeal swab samples in viral transport media at the icddr,b COVID-19 sample collection booth. All specimens and data collected by the staff-clinic were then sent to the Virology Laboratory of icddr,b for the SARS-CoV-2 rRT-PCR test. The Virology Laboratory also shared test results with the staff-clinic for further patient management.

### Ethical statements

The test and treatment of staff for COVID-19 was conducted under the icddr,b activity number ACT-01108 and ACT-01207; and this cohort study was conducted under protocol number PR-21065 and approved by the institutional review board of icddr,b. All participants provided informed consent during enrolment. In addition, all methods were performed in accordance with the relevant guidelines and regulations.

### COVID-19 detection

One aliquot of the collected nasopharyngeal swab was used for SARS-CoV-2 screening, and one was stored for future references. rRT-PCR was used to detect SARS-CoV-2 using RdRp (ORF1ab) and N gene-specific primers and probes^65^. The iTaq universal probes one-step kit (Bio-Rad Laboratories, CA, USA) was used in the Bio-Rad CFX96 Touch real-time PCR system. Threshold cycle (Ct) values of ≤ 37 were considered positive.

### Data management and analysis

Participants enrolled from 19 March 2020 to 31 March 2021 were included for the current study and were followed up until 31 July 2021. The baseline COVID-19 test data defined the cohort (Appendix 1B). Participants with negative rRT-PCR results during registration/baseline were followed up as a negative cohort. Participants with positive rRT-PCR results during registration or follow-up were considered a positive cohort and further followed-up for reinfection according to CDC guidelines for investigating the SARS-CoV-2 reinfection cases^14^. Participants who had two positive rRT-PCR tests 90 days apart were considered reinfection cases, including symptomatic cases testing positive ≥ 45 days after the first infection with paired respiratory specimens^14^. After each reinfection identification, an extended interview was conducted with a structured questionnaire in a Case Record Form. All data were recorded and analysed using SPSS (version 20). The proportion of each variable was analysed through chi-square or Fisher's exact test, where appropriate.

### SARS-CoV-2 genome sequencing and analysis

Besides our regular genomic surveillances^1^, reinfection cases were subjected to comparative genome characterisation; therefore, cases with Ct-values of ≤ 33 for both episodes (first infection and reinfection) were selected for whole-genome sequencing using the NGS method (Appendix 1C). All SARS-CoV-2 sequences under this study were submitted to publicly available online databases; GISAID (www.gisaid.org) and/or GenBank (www.ncbi.nlm.nih.gov/genbank/). Accession numbers are in Appendix 1D.

## Supplementary Information

Supplementary Information.

## Data Availability

The metadata and supplementary data will be available with the manuscript as a supplementary document in Appendix-1.
