# Supplementary material for: COVID-19 reinfections among naturally infected and vaccinated individuals
Source: Sci Rep. 2022 Jan 26;12:1438. doi: 10.1038/s41598-022-05325-5 (PMC8792012; doi:10.1038/s41598-022-05325-5)
Supplement: Supplementary file 1 — Supplementary Information. [file 41598_2022_5325_MOESM1_ESM.docx]

**APPENDIX-1**

**COVID-19 reinfections among naturally infected and vaccinated individuals**

Sezanur Rahman MSc^1^, M Mahfuzur Rahman MSc^1^, Mojnu Miah MSc^1^, Mst Noorjahan Begum PhD^1^, Monira Sarmin MCPS^2^, Mustafa Mahfuz MPH^2^, Mohammad Enayet Hossain PhD^1^, Mohammed Ziaur Rahman PhD^1^, Mohammod Jobayer Chisti PhD^2^, Tahmeed Ahmed PhD^2^, Shams El Arifeen PhD^3^, Mustafizur Rahman PhD^1^*

^1^Infectious Diseases Division, icddr,b: International Centre for Diarrhoeal Disease Research, Bangladesh, Mohakhali, Dhaka-1212, Bangladesh

^2^Nutrition and Clinical Services Division, icddr,b: International Centre for Diarrhoeal Disease Research, Bangladesh, Mohakhali, Dhaka-1212, Bangladesh

^3^Maternal and Child Health Division, icddr,b: International Centre for Diarrhoeal Disease Research, Bangladesh, Mohakhali, Dhaka-1212, Bangladesh

Correspondence to:

Mustafizur Rahman, 68 Shaheed Tajuddin Ahmed Sarani, Dhaka 1212, Bangladesh; mustafizur@icddrb.org; Telephone number: +880 2 9840523-32

**Table of contents**

| **Heading** | **Topics** | | **Page** |
| --- | --- | --- | --- |
| Appendix A | Evidence before this study | | 02 |
|  |  | MEDLINE (PubMed) search strategy,  Summary from the published cohort study,  Summary from the published case reports,  Summary from the published systematic review |  |
| Appendix B | Abbreviations and definitions | | 03 |
| Appendix C | Sequences Methodology | | 04 |
|  |  | Sequencing methods in brief  NGS sequences read raw data |  |
| Appendix D | Data in open access server | | 05 |
|  |  | Accession numbers of the SARS-CoV-2 sequencing from this study |  |
| Appendix E | Demographic data | | 06 |
|  |  | Table S1: Additional data (age, sex, blood group, date of infection and reinfection, Ct-value, date of vaccination, comorbidity, disease severity or outcome, and other risk factors) of re-infected cases. | 06 |
|  |  | Table S2: Additional data (symptoms during first infection and reinfection) of re-infected cases. | 08 |
| Appendix F | Other raw data | | 10 |
|  |  | Infections in person year calculation  Risk ratio analysis for co-morbidity of in-patient and outpatient group  Risk ratio analysis for reinfection  Risk ratio analysis for infection among vaccinated individuals  Comparative Ct-value (RdRp gene) analysis |  |
| Appendix G | Genomic surveillances | | 11 |
|  |  | Genomic surveillances data of icddr,b staff-clinic  Circulating variant in Dhaka |  |
| References | | | 12 |

**Appendix A: Evidence before this study**

**MEDLINE (PubMed) search strategy**

| **Search number** | **Search Details** | **Results** |
| --- | --- | --- |
| 1 | (sars*[Title]) OR (covid[Title]) | 149,678 |
| 2 | "reinfection*"[Title] OR "re infection*"[Title] OR "second episode"[Title] OR "recurrence*"[Title] OR "recrudescence*"[Title] OR "relapse*"[Title] | 76,395 |
| 3 | ((cohort[Title]) AND (search 1) AND (search 2) | 12 |
| 4 | (search 1) AND (search 2) with Filters: Case Reports published before 31 July 2021 | 121 |
| 5 | (search 1) AND (search 2) with Filters: Systematic Review | 7 |
|  |  |  |

**Summary from the published cohort study**

A total of 22 cohort studies were included after systematic search and references search. Among them, 16 cohort studies described recurrence of SARS-CoV-2 positive test after hospital discharge,^1-16^ presumed to be persistent viral shedding rather than reinfection, and six addressed reinfections. Three studies considered primary infection based on the baseline antibody test,^17-19^ increasing the risk of miscalculating reinfection as antibody responses depend on host immunity and time. Three studies considered rRT-PCR positive test ≥90 days apart for infection and reinfection identifications; however, two of them might overestimate the reinfection rate because they did not confer SARS-CoV-2 negative status between infection and reinfection,^20,21^ while patients may continue to shed virus and give rRT-PCR positive result even after 90 days. Only one cohort considered reinfection if rRT-PCR was positive ≥90 days after complete resolution of the first infection confirmed by ≥2 consecutive negative tests;^22^ however, this study did not perform genetic sequencing to verify reinfections.

**Summary from the published case reports**

Among systematically searched 121 case report articles, 38 did not align with our field of interest and were excluded after full-text read, and 32 articles describe the recurrence of SARS-CoV-2 positive that does not align with reinfection case definitions.^23-54^ A total of 79 cases described in 51 case report articles^55-105^ considered reinfection according to our reinfection case definitions, where only 14 cases from 12 articles were confirmed by whole-genome sequencing ^57,63,68,79,82,83,85,92-95,102^.

**Summary from the published systematic review**

The quality of the systematic review paper was accessed by considering three domains (i) methods for database search/ article filter criteria; (ii) type of included articles; and (iii) data extraction methods and quality of data. Finally, we considered 4 papers as good/ fair in quality. Among them, one paper indicates older age, moderate severity, bilateral pulmonary infiltration, decreased leucocytes, platelets, and CD4+T counts were associated with the recurrence of SARS-CoV-2.^106^ One study calculated 20 days mean-time for recurrence after discharge with increased disease severity in 2.3% cases,^107^ while another reported 18.8% increased severity for reinfection with genetically distinct SARS-CoV-2.^108^ A recent meta-analysis study included published literature up to 06 Mar 2021 and pooled estimation of confirmed reinfection was 0.3%.^109^

**Appendix B: Abbreviations and definitions**

**1. Abbreviations**

**95%CI** 95% Confidence interval

**ARDS** Acute Respiratory Distress Syndrome

**BP** Blood pressure

**COPD** Chronic obstructive pulmonary disease

**CRF** Case report form

**Ct** Threshold cycle

**HFNC** High Flow Nasal Cannula

**icddr,b** International Centre for Diarrhoeal Disease Research, Bangladesh

**ICU** Intensive care unit

**IQR** Interquartile range

**NGS** Next genome sequencing

**ORF** Open reading frames

**PCR** Polymerase chain reaction

**RdRp** RNA-dependent RNA polymerase

**RR** risk ratio

**rRT-PCR** Real-time reverse transcriptase polymerase chain reaction

**2. Definitions**

**Negative cohort:** Cohort with individuals, who were tested rRT-PCR negative for SARS-CoV-2 during enrolment and followed up*.

**Positive cohort:** Cohort with individuals, who were tested rRT-PCR positive for SARS-CoV-2 during enrolment or in negative cohort follow-up*.

**In-patient group:** COVID-19 patient, who were hospitalized and received treatment from icddr,b staff-clinic.

**Out-patient group:** COVID-19 patient, who received medication support from icddr,b staff-clinic but were not hospitalized.

**Reinfection:** ((Two PCR positive episodes, which are at least 90 days apart) **OR**

(Two PCR positive episodes, which are <90 days apart; if second episode was symptomatic with Ct-value <35)) **AND** ((there were ≥2 consecutive rRT-PCR negative test at 24 hours intervals between two episode) **OR** (supported by viral genomic data, such as the second episode showing phylogeny congruent with contemporaneous infections))).

**Episode 1:** First time infection either during enrolment or in follow-up.

**Episode 2:** Reinfection event (second infection) during follow-up. Positive result after episode 1 which were not true reinfection were excluded*.

*****After enrolment, any event of negative test results was excluded during data analysis.

**Appendix C: Sequences Methodology**

**Sequencing methods in brief**

RNA was extracted from archived nasopharyngeal samples using a QIAamp viral RNA Mini Kit (Qiagen, Hilden, Germany) according to the manufacturer’s protocol. Complementary DNA was obtained using the LunaScript^®^ RT SuperMix Kit (New England Biolabs^®^) and the whole-genome was subsequently amplified using ARTIC nCoV-2019 V3 primer panel in two different PCR pools by Q5^®^ Hot Start High-Fidelity 2X Master Mix. After dilution and merging two pools, Nanopore sequencing libraries were prepared following nCoV-2019 sequencing protocol v3 (LoCost) ^110^. Briefly, pooled amplicons were subjected to end-prep reaction, and end-prepped amplicons were taken forward to the native barcode ligation step (native barcode expansion packs EXP-NBD104 and EXP-NBD114). A batch of 24 barcoded amplicons was pooled, and a 0.4x Ampure XP bead (Beckman Coulter, California, USA) purification was carried out following the manufacturer’s protocol. The purified library pool was quantified by the Qubit 1x dsDNA High Sensitivity Assay Kit (Invitrogen) with a Qubit fluorometer (Invitrogen). The final library was quantified after sequencing adapter ligation with ONT Adapter Mix II (AMII) and 1x Ampure XP bead purification. Approximately 45 ng final libraries were loaded on the FLO-MIN106D (R9.4.1) flow cell on an Oxford Nanopore MinION MK 1C platform for 8 hours.

In total, 3,194,013 FASTQ reads (average length 489 bp) were generated by real-time base-calling with Guppy 4.3.4 as released with MinKNOW software with the fast base-calling mode. FASTQ reads were quality-checked, demultiplexed, trimmed, and a consensus FASTA file was generated using FASTQ QC + ARTIC + Nextclade r1.0.4 workflow on the cloud-based analysis platform ‘EPI2ME’ desktop agent v3.3.0 with default parameters. The consensus genomes and metadata were submitted to GISAID and/or GenBank database.

**NGS sequences read raw data**

| **Case Number** | **Episode 1 (E1)** | | | | **Corresponded Episode 2 (E2)** | | | |
| --- | --- | --- | --- | --- | --- | --- | --- | --- |
|  | **Strain name** | **Fastq Reads** | **Coverage (%)** | **GC content (%)** | **Strain same** | **Fastq Reads** | **Coverage (%)** | **GC content (%)** |
| Case-08 | icddrb-4615 | 44,964 | 69.71 | 38.4 | icddrb-5758 | 108,584 | 99.60 | 38 |
| Case-09 | icddrb-3083 | 82,113 | 95.19 | 38.1 | icddrb-5850 | 108,156 | 94.02 | 38.1 |
| Case-10 | icddrb-2798 | 95,359 | 94.34 | 38.1 | icddrb-5870 | 304,508 | 99.60 | 38 |
| Case-12 | icddrb-4107 | 30,293 | 91.43 | 38.1 | icddrb-6199 | 100,019 | 99.60 | 38 |
| Case-17 | icddrb-4896 | 23,013 | 95.19 | 38.1 | icddrb-7452 | 409,268 | 98.87 | 38 |
| Case-20 | icddrb-4349 | 45,463 | 95.07 | 38.1 | icddrb-7746 | 374,146 | 98.87 | 38 |
| Case-21 | icddrb-5743 | 48,197 | 96.03 | 38 | icddrb-7790 | 376,665 | 98.87 | 38 |
| Case-24 | icddrb-6037 | 32,107 | 91.82 | 38.1 | icddrb-7992 | 248,729 | 98.87 | 38 |
| Case-26 | icddrb-3357 | 61,176 | 84.15 | 38 | icddrb-8178 | 104,940 | 98.87 | 38 |
| Case-27 | icddrb-4037 | 61,205 | 94.16 | 38.1 | icddrb-8497A | 141,636 | 98.61 | 38 |
| Case-28 | icddrb-7738 | 234,206 | 98.87 | 38 | icddrb-8617 | 39,152 | 87.62 | 38 |
| Case-30 | icddrb-1475 | 54,467 | 62.17 | 38.4 | icddrb-8753 | 138,698 | 97.69 | 38 |
| Case-31 | icddrb-7707 | 155,110 | 98.87 | 38 | icddrb-8772 | 151,482 | 98.68 | 38 |
| Case-32 | icddrb-3867 | 147,435 | 99.60 | 38 | icddrb-8793 | 149,923 | 97.57 | 38 |
| Case-34 | icddrb-8087 | 178,554 | 98.87 | 38 | icddrb-8822 | 77,112 | 99.60 | 38 |
| Case-36 | icddrb-6441 | 206,411 | 99.58 | 38 | icddrb-9001 | 278,198 | 99.45 | 38 |
| Case-37 | icddrb-1989 | 193,987 | 99.60 | 38 | icddrb-9028 | 344,359 | 99.60 | 38 |

**Appendix D: Data in open access server**

**Accession numbers of the SARS-CoV-2 sequencing from this study**

| **Case No** | **GISAID EpiCoV^TM^ Accession ID*** | |
| --- | --- | --- |
|  | **Episode 1** | **Episode 2** |
| Case-08 | EPI_ISL_3506345 | EPI_ISL_3506350 |
| Case-09 | EPI_ISL_2361889 | EPI_ISL_2361901 |
| Case-10 | EPI_ISL_2361888 | EPI_ISL_2361900 |
| Case-12 | EPI_ISL_2361886 | EPI_ISL_2361898 |
| Case-16 | MW785206* | MW785207* |
| Case-17 | EPI_ISL_2361885 | EPI_ISL_2361897 |
| Case-19 |  | EPI_ISL_1715160 |
| Case-20 | EPI_ISL_2361883 | EPI_ISL_2361895 |
| Case-21 | EPI_ISL_2361882 | EPI_ISL_2361894 |
| Case-24 | EPI_ISL_2361880 | EPI_ISL_2361892 |
| Case-26 | EPI_ISL_3506338 | EPI_ISL_3506346 |
| Case-27 | EPI_ISL_3506339 | EPI_ISL_3506347 |
| Case-28 | EPI_ISL_3506340 | EPI_ISL_3506348 |
| Case-30 | EPI_ISL_3506341 | EPI_ISL_3611014 |
| Case-31 | EPI_ISL_3506342 | EPI_ISL_3611015 |
| Case-32 | EPI_ISL_3506343 | EPI_ISL_3611016 |
| Case-34 | EPI_ISL_3506344 | EPI_ISL_3506349 |
| Case-36 | EPI_ISL_3506353 | EPI_ISL_3506355 |
| Case-37 | EPI_ISL_3506352 | EPI_ISL_3506354 |

* GenBank Accession ID

**Appendix E: Demographic data**

**Table S1: Additional data (age, sex, blood group, date of infection and reinfection, Ct-value, date of vaccination, comorbidity, disease severity or outcome, and other risk factors) of re-infected cases.**

| **Case number** | **Age** | **Sex** | **Blood group^1^** | **Episode 1 (E1)** | | | **Episode 2 (E2)** | | | **Day Interval** | **Date of first vaccination^2^** | **Co-Morbidity^3^** | | | | | | **Other Risk Factor^3^** | | | | | |
| --- | --- | --- | --- | --- | --- | --- | --- | --- | --- | --- | --- | --- | --- | --- | --- | --- | --- | --- | --- | --- | --- | --- | --- |
|  |  |  |  |  |  |  |  |  |  |  |  |  |  |  |  |  |  | **E1** | | | **E2** | | |
|  |  |  |  | **Date** | **Ct-Value (RdRp)** | **Ct-Value (N)** | **Date** | **Ct-Value (RdRp)** | **Ct-Value (N)** |  |  | **Obesity** | **Diabetes** | **Asthma** | **Heart Disease** | **Lung Disease** | **High BP** | **Travel^4^** | **COVID Contact^5^** | **Hospital Visit^6^** | **Travel^4^** | **COVID Contact^5^** | **Hospital Visit^6^** |
| Case-01 | 46 | M | O+ | 30-May-20 | 34.5 |  | 16-Aug-20 | 25.0 | 24.8 | 78 |  | Y |  |  |  |  | Y |  |  |  |  | Y | Y |
| Case-02 | 35 | M | O+ | 3-Jun-20 | 16.1 |  | 1-Sep-20 | 33.2 | 33.7 | 90 |  | Y |  | Y |  |  |  |  |  |  |  | Y |  |
| Case-03 | 49 | F | B+ | 23-May-20 | 21.7 |  | 1-Sep-20 | 32.7 | 33.5 | 101 |  |  |  | Y |  |  |  |  |  | Y | Y |  |  |
| Case-04 | 39 | M | A+ | 16-May-20 | 37.0 |  | 4-Sep-20 | 34.8 | 34.9 | 111 |  |  |  |  |  |  | Y |  |  |  |  |  |  |
| Case-05 | 38 | M | AB- | 30-May-20 | 18.5 |  | 6-Sep-20 | 31.9 | 30.6 | 99 |  |  | Y |  |  |  |  |  | Y |  |  |  |  |
| Case-06 | 52 | F | B+ | 19-Jun-20 | 35.1 |  | 8-Sep-20 | 33.9 | 33.5 | 81 |  | Y | Y | Y |  |  | Y |  | Y |  |  | Y |  |
| Case-07 | 34 | F | B+ | 12-May-20 | 25.3 |  | 16-Sep-20 | 37.0 | 36.6 | 127 |  | Y | Y |  |  |  | Y |  |  |  |  |  |  |
| Case-08 | 39 | M | O+ | 6-Sep-20 | 35.5 | 33.4 | 23-Oct-20 | 14.9 | 14.8 | 47 |  |  |  |  |  |  |  |  | Y |  |  |  |  |
| Case-09 | 32 | M | B+ | 30-Jun-20 | 30.7 | 28.5 | 27-Oct-20 | 36.9 | 37.0 | 119 |  |  |  |  |  | Y |  |  |  |  |  |  |  |
| Case-10 | 45 | F | AB+ | 23-Jun-20 | 36.9 | 34.5 | 28-Oct-20 | 14.5 | 15.6 | 127 |  |  |  |  |  |  |  |  |  |  |  |  |  |
| Case-11 | 38 | F |  | 14-Sep-20 | 36.4 | 33.5 | 7-Nov-20 | 19.2 | 19.3 | 54 |  |  |  |  |  |  |  |  |  |  |  |  |  |
| Case-12 | 53 | M | A+ | 13-Aug-20 | 35.2 | 32.7 | 13-Nov-20 | 31.3 | 32.2 | 92 |  |  |  | Y |  |  |  |  | Y |  |  | Y |  |
| Case-13 | 58 | M | A+ | 2-Jun-20 | 30.3 | 33.1 | 8-Dec-20 | 21.4 | 21.8 | 189 |  |  |  |  |  | Y | Y |  |  |  |  |  |  |
| Case-14 | 28 | F |  | 21-Jul-20 | 25.5 | 26.2 | 8-Dec-20 | 29.2 | 30.3 | 140 |  |  |  |  |  |  |  |  |  |  |  |  |  |
| Case-15 | 56 | M | O- | 29-Aug-20 | 34.7 | 34.1 | 15-Dec-20 | 25.1 | 25.8 | 108 |  |  |  |  |  | Y |  |  |  |  |  | Y |  |
| Case-16 | 52 | M |  | 30-Nov-20 | 24.9 | 24.3 | 23-Feb-21 | 29.8 | 27.6 | 85 |  |  |  |  | Y |  |  |  | Y | Y |  | Y | Y |
| Case-17 | 53 | M |  | 16-Sep-20 | 36.4 | 33.3 | 9-Mar-21 | 15.9 | 16.2 | 174 |  |  |  |  |  |  |  |  |  |  |  |  |  |
| Case-18 | 45 | M |  | 20-Dec-20 | 13.5 | 13.5 | 24-Mar-21 | 33.7 | 33.0 | 94 | 15-Feb-21 |  |  |  |  |  |  | Y |  |  |  | Y |  |
| Case-19 | 59 | M |  | 30-May-20 | 37.0 | 36.2 | 25-Mar-21 | 21.1 | 20.2 | 299 | 16-Feb-21 |  |  |  |  |  |  |  |  |  |  |  |  |
| Case-20 | 25 | M |  | 26-Aug-20 | 36.5 | 34.1 | 30-Mar-21 | 21.7 | 21.6 | 216 |  |  |  |  |  |  |  |  |  |  |  |  |  |
| Case-21 | 55 | M | B- | 22-Oct-20 | 36.3 | 31.9 | 1-Apr-21 | 19.4 | 18.7 | 161 |  |  |  |  | Y |  |  |  |  |  |  | Y |  |
| Case-22 | 48 | M |  | 20-Aug-20 | 11.9 | 14.2 | 4-Apr-21 | 36.0 | 32.2 | 227 | 14-Feb-21 |  |  |  |  |  |  |  |  |  |  |  |  |
| Case-23 | 31 | F |  | 24-Jun-20 | 24.3 | 22.5 | 4-Apr-21 | 36.9 | 34.3 | 284 | 17-Feb-21 |  |  |  |  |  |  |  | Y |  |  |  |  |
| Case-24 | 50 | M | B+ | 5-Nov-20 | 34.6 | 32.1 | 6-Apr-21 | 15.2 | 16.4 | 152 | 3-Mar-21 |  | Y |  |  |  | Y |  |  |  |  |  |  |
| Case-25 | 33 | F | AB+ | 15-Sep-20 | 36.9 | 31.2 | 7-Apr-21 | 23.4 | 23.0 | 204 |  | Y |  | Y |  |  |  |  | Y |  |  | Y |  |
| Case-26 | 31 | F | A+ | 9-Jul-20 | 36.1 | 34.1 | 14-Apr-21 | 27.4 | 26.6 | 279 | 28-Feb-21 |  | Y |  |  |  |  |  |  |  |  |  |  |
| Case-27 | 29 | F | B+ | 9-Aug-20 | 33.9 | 33.2 | 4-Jun-21 | 24.2 | 22.5 | 299 |  |  |  |  |  |  |  |  |  | Y |  | Y |  |
| Case-28 | 28 | M | A+ | 30-Mar-21 | 20.4 | 19.7 | 22-Jun-21 | 33.4 | 31.4 | 83 |  |  |  |  |  |  |  |  |  | Y |  |  | Y |
| Case-29 | 27 | M | O+ | 29-Nov-20 | 36.8 | 35.3 | 25-Jun-21 | 16.2 | 15.5 | 217 | 28-Feb-21* |  |  |  |  |  |  |  |  |  |  |  |  |
| Case-30 | 33 | M | B- | 28-May-20 | 33.1 | 32.4 | 1-Jul-21 | 14.0 | 12.0 | 399 | 13-Mar-21* |  |  |  |  |  |  |  |  |  |  |  |  |
| Case-31 | 31 | M | A+ | 29-Mar-21 | 18.1 | 17.4 | 2-Jul-21 | 17.0 | 16.6 | 460 |  |  |  |  |  |  | Y | Y |  | Y | Y |  | Y |
| Case-32 | 32 | M | O+ | 29-Jul-20 | 26.7 | 24.1 | 3-Jul-21 | 18.3 | 19.0 | 339 |  |  |  |  |  |  |  |  |  | Y |  | Y | Y |
| Case-33 | 33 | M | A+ | 28-Jul-20 | 19.1 | 19.0 | 3-Jul-21 | 29.6 | 31.2 | 340 |  |  |  |  |  |  |  |  |  |  |  |  |  |
| Case-34 | 30 | M | O+ | 9-Apr-21 | 15.1 | 15.2 | 5-Jul-21 | 32.1 | 30.3 | 87 |  |  |  |  |  |  |  |  |  | Y |  | Y | Y |
| Case-35 | 29 | M | B+ | 8-Jul-20 | 17.3 | 16.9 | 15-Jul-21 | 16.9 | 17.2 | 372 |  |  |  |  |  |  |  |  |  |  |  | Y |  |
| Case-36 | 56 | M | B+ | 23-Nov-20 | 24.4 | 23.3 | 16-Jul-21 | 18.4 | 17.8 | 235 |  |  | Y |  |  |  | Y |  | Y | Y |  |  |  |
| Case-37 | 30 | M | O+ | 4-Jun-20 | 28.5 | 28.8 | 17-Jul-21 | 15.2 | 14.0 | 408 |  |  |  |  |  |  |  |  |  |  |  |  |  |
| Case-38 | 43 | F | A+ | 1-May-20 | 27.6 | 29.3 | 29-Jul-21 | 14.9 | 12.0 | 454 | 8-Mar-21* |  |  | Y |  |  |  |  |  | Y |  | Y |  |

^1^ If available in the database; ^2^ If vaccinated before reinfection (* completed two doses vaccination); ^3^ Fill colour denote Yes; ^4^ In different district using public transport; ^5^ In contact with another COVID-19 patient; ^6^ In-patient or out-patient treatment facility whether as for medical duty/ seeking treatment/ visiting another patient. [Note: Case no 11 and 14 refused to participate in extended interview after re-infected]

**Table S2: Additional data (symptoms during first infection and reinfection) of re-infected cases.**

| **Case number** | **Episode 1 (E1)** | | | | | | | | | | | | | | | | | | | **Episode 2 (E2)** | | | | | | | | | | | | | | | | | |
| --- | --- | --- | --- | --- | --- | --- | --- | --- | --- | --- | --- | --- | --- | --- | --- | --- | --- | --- | --- | --- | --- | --- | --- | --- | --- | --- | --- | --- | --- | --- | --- | --- | --- | --- | --- | --- | --- |
|  | **Date onset^1^** | **Symptoms^2^** | | | | | | | | | | | | | | | **Care^2^** | | | **Date onset^1^** | **Symptoms^2^** | | | | | | | | | | | | | | **Care^2^** | | |
|  |  | **Fever** | **Sore throat** | **Cough** | **SOB** | **Vomiting** | **Diarrhoea** | **Headache** | **Conjunctivitis** | **Muscle aches** | **Joint pain** | **Loss of appetite** | **Weakness** | **Irritation in body** | **Asthmatic problem** | **Congestion** | **Hospitalization** | **Medication^3^** | **Antibiotic** |  | **Fever** | **Sore throat** | **Cough** | **SOB** | **Vomiting** | **Diarrhoea** | **Headache** | **Conjunctivitis** | **Muscle aches** | **Joint** | **Appetite** | **Consciousness** | **Weakness** | **Asthmatic problem** | **Hospitalization** | **Medication^3^** | **Antibiotic** |
| Case-01 | 28.05.20 |  |  |  |  |  |  | y |  |  |  |  |  |  |  |  |  |  |  | 12.08.20 | y |  | y | y |  |  |  |  |  |  |  |  | y |  |  | y | y |
| Case-02 | 01.06.20 |  |  |  | y | y | y |  |  |  |  | y | y |  | y |  |  | y | y | 29.08.20 |  |  |  | y |  | y |  |  |  |  |  |  |  |  |  |  |  |
| Case-03 | 19.05.20 |  |  |  |  |  |  |  |  |  |  |  |  |  | y |  |  |  |  | 27.08.20 | y |  | y |  |  | y |  |  | y |  |  |  | y |  |  | y | y |
| Case-04 | 13.05.20 |  | y |  |  |  |  |  |  |  |  |  |  |  | y |  |  | y |  | 02.09.20 | y |  |  |  |  |  |  |  |  |  |  |  |  | y |  |  |  |
| Case-05 |  |  |  |  |  |  |  |  |  |  |  |  |  |  |  |  |  |  |  | 03.09.20 | y |  | y |  |  |  |  |  |  |  |  |  |  |  |  | y |  |
| Case-06 | 17.06.20 |  |  |  |  |  |  |  |  |  |  |  |  |  |  |  |  |  |  | 06.09.20 |  |  |  |  |  |  |  |  |  |  |  |  |  |  |  |  |  |
| Case-07 | 07.05.20 |  |  |  |  |  |  |  |  |  |  |  |  |  |  |  |  |  |  | 18.09.20 |  |  |  |  |  |  | y |  |  |  |  |  |  |  |  |  |  |
| Case-08 |  |  |  |  |  |  |  |  |  |  |  |  |  |  |  |  |  |  |  | 20.10.20 | y |  | y |  |  | y |  |  | y |  |  |  | y |  |  | y | y |
| Case-09 | 27.05.20 |  |  | y |  |  |  |  |  |  |  |  |  |  | y |  |  |  |  | 31.10.20 |  | y | y |  |  |  |  |  |  |  |  |  | y |  |  | y |  |
| Case-10 |  |  |  |  |  |  |  |  |  |  |  |  |  |  |  |  |  |  |  | 23.10.20 | y |  |  |  |  |  |  |  |  |  |  |  |  |  |  | y |  |
| Case-12 | 11.08.20 |  |  | y | y |  |  |  |  |  |  |  | y |  |  |  |  | y | y |  |  |  |  |  |  |  |  |  |  |  |  |  |  |  |  |  |  |
| Case-13 | 01.06.20 |  |  | y |  |  |  |  |  |  |  |  |  |  |  |  |  | y | y | 14.12.20 |  |  | y |  |  |  |  |  |  |  | y |  |  |  |  |  |  |
| Case-15 | 21.08.20 |  |  | y |  |  | y |  |  | y | y | y | y |  |  |  |  | y | y | 12.12.20 |  | y |  |  | y |  | y |  | y | y |  |  | y |  |  | y |  |
| Case-16 | 28.11.20 |  | y | y |  |  |  |  |  |  |  |  | y |  |  |  |  | y | y | 21.02.21 | y | y | y |  |  |  | y |  |  |  |  |  | y |  | y | y | y |
| Case-17 | 14.09.20 |  |  |  |  |  |  |  |  |  |  |  |  |  |  | y |  |  |  | 07.03.21 | y |  | y |  |  |  |  |  |  |  | y |  |  |  |  | y |  |
| Case-18 | 18.12.20 |  |  |  |  |  |  |  |  | y |  | y |  |  |  |  |  | y |  | 23.03.21 |  |  |  |  |  |  |  |  | y |  | y |  | y |  |  |  |  |
| Case-19 |  |  |  |  |  |  |  |  |  |  |  |  |  |  |  |  |  |  |  | 23.03.21 | y |  | y |  |  | y |  |  | y |  | y |  |  |  |  | y | y |
| Case-20 | 24.08.20 |  | y | y |  |  |  |  | y |  |  |  |  |  |  |  |  |  |  | 28.03.21 | y |  |  |  |  |  | y |  | y |  |  |  |  |  |  |  |  |
| Case-21 | 18.10.20 |  |  | y |  |  |  |  |  |  |  |  |  |  |  |  |  | y |  | 31.03.21 |  | y | y |  |  | y | y |  |  |  |  |  |  |  |  | y | y |
| Case-22 | 18.08.20 |  | y |  |  |  |  |  |  |  |  |  |  |  |  | y |  |  |  | 01.04.21 | y | y | y |  |  | y |  |  |  |  |  |  |  |  |  | y |  |
| Case-23 | 22.06.20 |  |  | y |  |  |  |  |  |  |  |  |  | y |  |  |  | y |  | 01.04.21 | y |  | y |  |  | y | y |  |  |  |  |  | y |  |  | y |  |
| Case-24 | 19.10.20 |  |  | y |  | y |  | y |  |  |  |  |  |  |  |  |  | y | y | 04.04.21 | y |  | y |  | y |  | y |  |  |  |  |  |  | y |  | y | y |
| Case-25 | 12.09.20 |  | y | y |  |  |  |  |  |  |  |  |  |  | y |  | y | y | y | 02.04.21 | y | y | y |  | y |  |  |  | y |  |  |  | y | y |  | y | y |
| Case-26 | 07.07.20 |  |  |  |  |  |  | y |  |  |  |  |  |  |  |  |  |  |  | 13.04.21 | y |  | y |  |  |  |  |  |  |  |  |  |  |  |  | y |  |
| Case-27 |  |  |  |  |  |  |  |  |  |  |  |  |  |  |  |  |  |  |  | 02.06.21 | y | y | y |  |  |  | y |  |  |  | y |  | y | y |  | y | y |
| Case-28 | 26.03.21 |  |  | y |  |  |  |  |  |  |  |  |  |  |  |  |  | y |  | 20.06.21 | y |  |  |  |  |  |  | y |  |  |  |  |  | y |  |  |  |
| Case-29 | 27.11.20 |  | y | y |  |  |  |  |  |  |  |  | y | y |  |  |  | y | y | 23.06.20 | y |  | y |  |  |  |  |  |  |  |  |  |  |  |  | y |  |
| Case-30 | 25.05.20 |  | y | y |  |  |  |  |  |  |  |  |  |  |  |  |  |  |  | 30.06.21 | y |  |  |  |  |  | y |  | y |  |  |  |  |  |  |  |  |
| Case-31 | 27.03.21 |  | y | y |  |  |  |  |  |  |  |  | y |  |  |  |  |  |  | 01.07.21 | y | y | y |  |  |  | y |  |  |  |  |  | y |  |  | y |  |
| Case-32 | 28.07.21 |  |  | y |  |  |  | y |  | y |  |  | y |  |  |  |  | y | y | 01.07.21 |  | y | y |  | y |  | y |  |  |  |  | y | y |  |  | y | y |
| Case-33 | 27.07.20 |  |  | y |  |  |  |  |  | y |  |  |  |  |  |  |  | y |  | 02.07.20 | y | y | y |  |  |  | y |  |  | y |  |  | y |  |  | y | y |
| Case-34 | 07.04.21 |  | y | y |  |  |  |  |  |  |  |  |  |  |  |  |  | y | y | 04.07.21 | y |  |  |  |  |  | y |  |  |  |  |  | y |  |  |  |  |
| Case-35 | 08.07.20 |  |  |  |  |  |  | y |  |  |  | y |  |  |  |  |  | y | y | 14.07.21 | y |  |  |  |  |  |  |  |  |  |  |  | y |  |  | y | y |
| Case-36 | 22.11.20 |  |  |  |  |  |  | y |  |  |  | y | y |  |  |  |  | y | y | 15.07.21 | y |  |  |  |  |  |  |  |  |  |  |  |  |  |  | y | y |
| Case-37 | 02.06.20 |  |  |  |  |  |  |  |  |  |  |  |  |  |  |  |  | y |  | 16.07.21 | y |  |  |  |  |  |  |  |  | y |  |  |  |  |  | y |  |
| Case-38 | 30.04.20 |  | y | y | y |  |  |  |  |  |  |  |  |  |  |  |  | y | y | 25.07.21 | y | y | y |  |  |  |  |  |  |  |  |  |  |  |  | y | y |

^1^ The asymptomatic case remained blank; ^2^ Fill colour denote Yes; ^3^ Other than conventional medication recommended by COVID-19 clinical management guideline;

**Appendix F: Other raw data**

**F1. Infections in person year calculation**

|  | Negative cohort | Positive Cohort |
| --- | --- | --- |
| Total infection | 277 | 38 |
| Total person-year | 1074.24 | 667.19 |
| Person-year | 0.995 | 0.91 |
| Infections in 100 person-year | 25.78 | 5.70 |

**F2. Risk ratio analysis for co-morbidity of in-patient and outpatient group**

|  | In-Patient | Out-patient | Risk ratio | 95% CI | p-value* |
| --- | --- | --- | --- | --- | --- |
| Hypertension | 94 | 71 | 1.381 | 1.138 ̶ 1.677 | 0.001 |
| Not-present | 113 | 161 |  |  |  |
| Diabetes mellitus | 73 | 43 | 1.517 | 1.254 ̶ 1.835 | <0.001 |
| Not-present | 134 | 189 |  |  |  |
| Asthma/COPD | 33 | 27 | 1.198 | 0.930 ̶ 1.544 | 0.19 |
| Not-present | 174 | 205 |  |  |  |

* Mantel-Haenszel chi square (2-tail)

**F3. Risk ratio analysis for reinfection**

|  | Positive cohort | Negative cohort | Risk ratio | 95% CI | p-value* |
| --- | --- | --- | --- | --- | --- |
| Infected | 38 | 277 | 0.257 | 0.190 ̶ 0.347 | <0.001 |
| Not infected | 713 | 803 |  |  |  |

* Mantel-Haenszel chi square (2-tail)

**F4. Risk ratio analysis for infection among vaccinated individuals (1 May 2021- 31 Jul 2021)**

|  | Vaccinated^1^ | Not vaccinated | Risk ratio | 95% CI | p-value* |
| --- | --- | --- | --- | --- | --- |
| SARS-CoV-2 Positive | 37 | 96 | 1.08 | 0.76 ̶ 1.534 | 0.66 |
| SARS-CoV-2 Negative | 59 | 170 |  |  |  |

^1^ Individual received two-dose vaccination before infections

|  | Vaccinated  (Full dose) | Vaccinated  (One dose) | Risk ratio | 95% CI | p-value* |
| --- | --- | --- | --- | --- | --- |
| SARS-CoV-2 Positive | 38 | 66 | 1.096 | 0.51 ̶ 2.34 | 0.81 |
| SARS-CoV-2 Negative | 5 | 10 |  |  |  |

* Mantel-Haenszel chi square (2-tail)

**F5. Comparative Ct-value (RdRp gene) analysis**

|  |  | Mean | Median | IQR | p-value* |
| --- | --- | --- | --- | --- | --- |
| Episode 1 | Symptomatic cases | 22.93 | 20.44 | 17.00 ̶ 27.61 | 0.01 |
|  | Asymptomatic cases | 25.32 | 22.79 | 16.80 ̶ 35.11 |  |
|  | Cases that were not re-infected | 22.90 | 20.39 | 16.85 ̶ 28.16 | <0.001 |
|  | Re-infected cases | 29.65 | 34.54 | 20.44 ̶ 36.33 |  |
|  | Not Hospitalized cases | 23.42 | 20.68 | 16.90 ̶ 29.56 | 0.37 |
|  | Hospitalized cases | 22.44 | 20.97 | 16.33 ̶ 26.02 |  |
| Re-infected case | Episode 1 | 29.65 | 34.54 | 20.44 ̶ 36.33 | 0.1 |
|  | Episode 2 | 26.28 | 26.23 | 18.96 ̶ 33.27 |  |

* Independent samples t-Test (2-tail)

**Appendix G: Genomic surveillances**

**G1. Genomic surveillances data of icddr,b staff-clinic (16 Dec, 2020 to 31 Jul, 2021)**


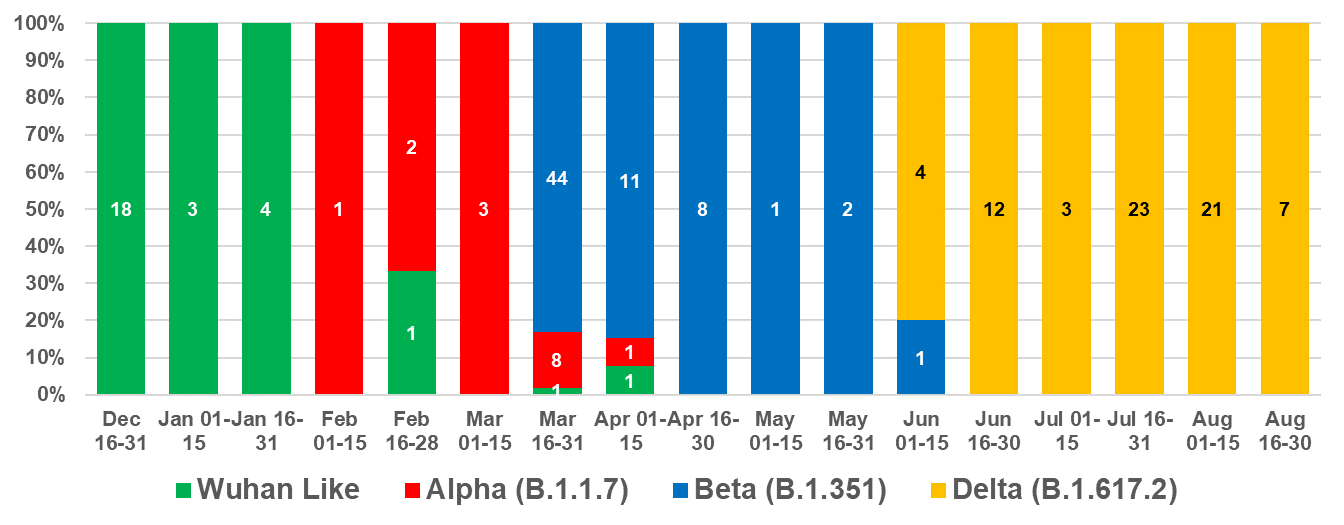


**G2. Circulating variant in Dhaka (01 May, 2021 to 31 Jul, 2021)**

| **Variant Name** | **Total number** | **%** |
| --- | --- | --- |
| Beta (B.1.351) | 35 | 13.6 |
| Nigeria (B.1.525) | 7 | 2.7 |
| Delta (B.1.617.2) | 214 | 83.3 |
| Other | 1 | 0.4 |
| **Total** | 257 | 100.0 |

**References**

1 Ao, Z. *et al.* Clinical characteristics and potential factors for recurrence of positive SARS-CoV-2 RNA in convalescent patients: a retrospective cohort study. *Clinical and experimental medicine* **21**, 361-367, doi:10.1007/s10238-021-00687-y (2021).

2 Chen, J. *et al.* Clinical course and risk factors for recurrence of positive SARS-CoV-2 RNA: a retrospective cohort study from Wuhan, China. *Aging* **12**, 16675-16689, doi:10.18632/aging.103795 (2020).

3 Chen, L. Z. *et al.* Can elevated concentrations of ALT and AST predict the risk of 'recurrence' of COVID-19? *Epidemiology and infection* **148**, e218, doi:10.1017/s0950268820002186 (2020).

4 Chen, S. L. *et al.* Epidemiological and Clinical Findings of Short-Term Recurrence of Severe Acute Respiratory Syndrome Coronavirus 2 Ribonucleic Acid Polymerase Chain Reaction Positivity in 1282 Discharged Coronavirus Disease 2019 Cases: A Multicenter, Retrospective, Observational Study. *Open forum infectious diseases* **7**, ofaa432, doi:10.1093/ofid/ofaa432 (2020).

5 Du, H. W. *et al.* Prevalence and outcomes of re-positive nucleic acid tests in discharged COVID-19 patients. *European journal of clinical microbiology & infectious diseases : official publication of the European Society of Clinical Microbiology* **40**, 413-417, doi:10.1007/s10096-020-04024-1 (2021).

6 He, S. *et al.* Positive RT-PCR Test Results in 420 Patients Recovered From COVID-19 in Wuhan: An Observational Study. *Frontiers in pharmacology* **11**, 549117, doi:10.3389/fphar.2020.549117 (2020).

7 Kang, Y. J. South Korea's COVID-19 Infection Status: From the Perspective of Re-positive Test Results After Viral Clearance Evidenced by Negative Test Results. *Disaster medicine and public health preparedness* **14**, 762-764, doi:10.1017/dmp.2020.168 (2020).

8 Li, Y. *et al.* Positive result of Sars-Cov-2 in faeces and sputum from discharged patients with COVID-19 in Yiwu, China. *Journal of medical virology* **92**, 1938-1947, doi:10.1002/jmv.25905 (2020).

9 Lu, J. *et al.* Clinical, immunological and virological characterization of COVID-19 patients that test re-positive for SARS-CoV-2 by RT-PCR. *EBioMedicine* **59**, 102960, doi:10.1016/j.ebiom.2020.102960 (2020).

10 Shui, T. J., Li, C., Liu, H. B., Chen, X. & Zhang, B. K. Characteristics of recovered COVID-19 patients with recurrent positive RT-PCR findings in Wuhan, China: a retrospective study. *BMC infectious diseases* **20**, 749, doi:10.1186/s12879-020-05463-z (2020).

11 Wu, J. *et al.* Coronavirus Disease 2019 Test Results After Clinical Recovery and Hospital Discharge Among Patients in China. *JAMA network open* **3**, e209759, doi:10.1001/jamanetworkopen.2020.9759 (2020).

12 Xiao, A. T., Tong, Y. X. & Zhang, S. False negative of RT-PCR and prolonged nucleic acid conversion in COVID-19: Rather than recurrence. *Journal of medical virology* **92**, 1755-1756, doi:10.1002/jmv.25855 (2020).

13 Yuan, B. *et al.* Recurrence of positive SARS-CoV-2 viral RNA in recovered COVID-19 patients during medical isolation observation. *Scientific reports* **10**, 11887, doi:10.1038/s41598-020-68782-w (2020).

14 Zheng, J. *et al.* Incidence, clinical course and risk factor for recurrent PCR positivity in discharged COVID-19 patients in Guangzhou, China: A prospective cohort study. *PLoS neglected tropical diseases* **14**, e0008648, doi:10.1371/journal.pntd.0008648 (2020).

15 Zhu, H. *et al.* Clinical features of COVID-19 convalescent patients with re-positive nucleic acid detection. *Journal of clinical laboratory analysis* **34**, e23392, doi:10.1002/jcla.23392 (2020).

16 Zou, Y. *et al.* The Issue of Recurrently Positive Patients Who Recovered From COVID-19 According to the Current Discharge Criteria: Investigation of Patients from Multiple Medical Institutions in Wuhan, China. *The Journal of infectious diseases* **222**, 1784-1788, doi:10.1093/infdis/jiaa301 (2020).

17 Hall, V. J. *et al.* SARS-CoV-2 infection rates of antibody-positive compared with antibody-negative health-care workers in England: a large, multicentre, prospective cohort study (SIREN). *Lancet (London, England)* **397**, 1459-1469, doi:10.1016/s0140-6736(21)00675-9 (2021).

18 Leidi, A. *et al.* Risk of reinfection after seroconversion to SARS-CoV-2: A population-based propensity-score matched cohort study. *Clinical infectious diseases : an official publication of the Infectious Diseases Society of America*, doi:10.1093/cid/ciab495 (2021).

19 Veronica, F., Anne, R., Christopher, B., Kenneth, C. & Jon, R. Incidence of COVID-19 recurrence among large cohort of healthcare employees. *Annals of epidemiology* **60**, 8-14, doi:10.1016/j.annepidem.2021.04.005 (2021).

20 Sheehan, M. M., Reddy, A. J. & Rothberg, M. B. Reinfection Rates among Patients who Previously Tested Positive for COVID-19: a Retrospective Cohort Study. *Clinical infectious diseases : an official publication of the Infectious Diseases Society of America*, doi:10.1093/cid/ciab234 (2021).

21 Slezak, J. *et al.* Rate and severity of suspected SARS-Cov2 reinfection in a cohort of PCR-positive COVID-19 patients. *Clinical microbiology and infection : the official publication of the European Society of Clinical Microbiology and Infectious Diseases*, doi:10.1016/j.cmi.2021.07.030 (2021).

22 Flacco, M. E. *et al.* Rate of reinfections after SARS-CoV-2 primary infection in the population of an Italian province: a cohort study. *Journal of public health (Oxford, England)*, doi:10.1093/pubmed/fdab346 (2021).

23 Abdallah, H., Porterfield, F. & Fajgenbaum, D. Symptomatic relapse and long-term sequelae of COVID-19 in a previously healthy 30-year-old man. *BMJ case reports* **13**, doi:10.1136/bcr-2020-239825 (2020).

24 Alonso, F. O. M., Sabino, B. D., Guimarães, M. & Varella, R. B. Recurrence of SARS-CoV-2 infection with a more severe case after mild COVID-19, reversion of RT-qPCR for positive and late antibody response: Case report. *Journal of medical virology* **93**, 655-656, doi:10.1002/jmv.26432 (2021).

25 Bellanti, F. *et al.* Fatal relapse of COVID-19 after recovery? A case report of an older Italian patient. *The Journal of infection* **82**, e49-e51, doi:10.1016/j.jinf.2020.12.009 (2021).

26 Bonifácio, L. P. *et al.* Are SARS-CoV-2 reinfection and Covid-19 recurrence possible? a case report from Brazil. *Revista da Sociedade Brasileira de Medicina Tropical* **53**, e20200619, doi:10.1590/0037-8682-0619-2020 (2020).

27 Chen, D. *et al.* Recurrence of positive SARS-CoV-2 RNA in COVID-19: A case report. *International journal of infectious diseases : IJID : official publication of the International Society for Infectious Diseases* **93**, 297-299, doi:10.1016/j.ijid.2020.03.003 (2020).

28 de Brito, C. A. A., Lima, P. M. A., de Brito, M. C. M. & de Oliveira, D. B. Second Episode of COVID-19 in Health Professionals: Report of Two Cases. *International medical case reports journal* **13**, 471-475, doi:10.2147/imcrj.s277882 (2020).

29 Dou, C. *et al.* A case presentation for positive SARS-CoV-2 RNA recurrence in a patient with a history of type 2 diabetes that had recovered from severe COVID-19. *Diabetes research and clinical practice* **166**, 108300, doi:10.1016/j.diabres.2020.108300 (2020).

30 Duggan, N. M., Ludy, S. M., Shannon, B. C., Reisner, A. T. & Wilcox, S. R. Is novel coronavirus 2019 reinfection possible? Interpreting dynamic SARS-CoV-2 test results. *The American journal of emergency medicine* **39**, 256.e251-256.e253, doi:10.1016/j.ajem.2020.06.079 (2021).

31 Gaire, D., Sah, M. & Singh, B. COVID-19 Reinfection in a Young Medical Doctor: A Case Report. *JNMA; journal of the Nepal Medical Association* **59**, 712-715, doi:10.31729/jnma.6450 (2021).

32 Gao, G. *et al.* Absent immune response to SARS-CoV-2 in a 3-month recurrence of coronavirus disease 2019 (COVID-19) case. *Infection* **49**, 57-61, doi:10.1007/s15010-020-01485-6 (2021).

33 He, F. *et al.* Successful recovery of recurrence of positive SARS-CoV-2 RNA in COVID-19 patient with systemic lupus erythematosus: a case report and review. *Clinical rheumatology* **39**, 2803-2810, doi:10.1007/s10067-020-05230-0 (2020).

34 Inada, M. *et al.* Asymptomatic COVID-19 re-infection in a Japanese male by elevated half-maximal inhibitory concentration (IC(50)) of neutralizing antibodies. *Journal of infection and chemotherapy : official journal of the Japan Society of Chemotherapy* **27**, 1063-1067, doi:10.1016/j.jiac.2021.04.017 (2021).

35 Jadeja, D. & Basak, P. Symptomatic Reinfection in Previously Recovered Coronavirus Disease 2019 (COVID-19) Geriatric Patient. *Cureus* **13**, e13961, doi:10.7759/cureus.13961 (2021).

36 Konstantinou, F. *et al.* A Case of SARS-CoV-2 Clinical Relapse after 4 Negative RT-PCR Tests in Greece: Recurrence or Reinfection? *Clinical medicine insights. Case reports* **14**, 11795476211009813, doi:10.1177/11795476211009813 (2021).

37 Lafaie, L. *et al.* Recurrence or Relapse of COVID-19 in Older Patients: A Description of Three Cases. *Journal of the American Geriatrics Society* **68**, 2179-2183, doi:10.1111/jgs.16728 (2020).

38 Leung, S. & Hossain, N. Recurrence and Recovery of COVID-19 in an Older Adult Patient with Multiple Comorbidities: A Case Report. *Gerontology* **67**, 445-448, doi:10.1159/000514675 (2021).

39 Liu, F. *et al.* Repeated COVID-19 relapse during post-discharge surveillance with viral shedding lasting for 67 days in a recovered patient infected with SARS-CoV-2. *Journal of microbiology, immunology, and infection = Wei mian yu gan ran za zhi* **54**, 101-104, doi:10.1016/j.jmii.2020.07.017 (2021).

40 Loh, S. Y., Bassett, J., Hoodless, E. J. & Walshaw, M. Possible COVID-19 reinfection in a patient with X-linked agammaglobulinaemia. *BMJ case reports* **14**, doi:10.1136/bcr-2020-240765 (2021).

41 Martín Enguix, D., Aguirre Rodríguez, J. C., Sánchez Cambronero, M. & Hidalgo Rodríguez, A. [PCR for COVID-19 positive, then negative and again positive Reinfection at 55 days?]. *Semergen* **47**, 207-208, doi:10.1016/j.semerg.2020.12.001 (2021).

42 Munoz Mendoza, J. & Alcaide, M. L. COVID-19 in a patient with end-stage renal disease on chronic in-center hemodialysis after evidence of SARS-CoV-2 IgG antibodies. Reinfection or inaccuracy of antibody testing. *IDCases* **22**, e00943, doi:10.1016/j.idcr.2020.e00943 (2020).

43 Nouira, N. *et al.* Relapse of rare diseases during COVID-19 pandemic: bicytopenia in an adult patient with thiamine-responsive megaloblastic anaemia. *The Pan African medical journal* **35**, 139, doi:10.11604/pamj.supp.2020.35.139.25368 (2020).

44 Okar, L., Ahmad, R. & Yassin, M. A. First report of COVID-19 reinfection in a patient with beta thalassemia major. *Clinical case reports* **9**, 861-865, doi:10.1002/ccr3.3682 (2021).

45 Ormazabal Vélez, I. *et al.* Two patients with rituximab associated low gammaglobulin levels and relapsed covid-19 infections treated with convalescent plasma. *Transfusion and apheresis science : official journal of the World Apheresis Association : official journal of the European Society for Haemapheresis* **60**, 103104, doi:10.1016/j.transci.2021.103104 (2021).

46 Palomba, E. *et al.* Treatment of SARS-CoV-2 relapse with remdesivir and neutralizing antibodies cocktail in a patient with X-linked agammaglobulinaemia. *International journal of infectious diseases : IJID : official publication of the International Society for Infectious Diseases* **110**, 338-340, doi:10.1016/j.ijid.2021.07.064 (2021).

47 Parekh, Y. H., Altomare, N. J., McDonnell, E. P., Blaser, M. J. & Parikh, P. D. Recurrence of Upper Extremity Deep Vein Thrombosis Secondary to COVID-19. *Viruses* **13**, doi:10.3390/v13050878 (2021).

48 Reuken, P. A. *et al.* Severe clinical relapse in an immunocompromised host with persistent SARS-CoV-2 infection. *Leukemia* **35**, 920-923, doi:10.1038/s41375-021-01175-8 (2021).

49 Romera, I. *et al.* SARS-CoV-2 reinfection. *Medicina intensiva* **45**, 375-376, doi:10.1016/j.medine.2021.04.009 (2021).

50 Roy, S. COVID-19 Reinfection in the Face of a Detectable Antibody Titer. *Cureus* **13**, e14033, doi:10.7759/cureus.14033 (2021).

51 Tanaka, A. *et al.* Development of toxic epidermal necrolysis in a coronavirus disease 2019 patient with recurrence of positive SARS-CoV-2 viral RNA. *The Journal of dermatology* **48**, e144-e145, doi:10.1111/1346-8138.15753 (2021).

52 Wu, J. *et al.* Recurrence of SARS-CoV-2 nucleic acid positive test in patients with COVID-19: a report of two cases. *BMC pulmonary medicine* **20**, 308, doi:10.1186/s12890-020-01348-8 (2020).

53 Zaffina, S. *et al.* Recurrence, Reactivation, or Inflammatory Rebound of SARS-CoV-2 Infection With Acute Vestibular Symptoms: A Case Report and Revision of Literature. *Frontiers in human neuroscience* **15**, 666468, doi:10.3389/fnhum.2021.666468 (2021).

54 Zhang, R. Z. *et al.* Case Report: Recurrence of Positive SARS-CoV-2 Results in Patients Recovered From COVID-19. *Frontiers in medicine* **7**, 585485, doi:10.3389/fmed.2020.585485 (2020).

55 Ahmadian, S., Fathizadeh, H., Shabestari Khiabani, S., Asgharzadeh, M. & Kafil, H. S. COVID-19 reinfection in a healthcare worker after exposure with high dose of virus: A case report. *Clinical case reports* **9**, e04257, doi:10.1002/ccr3.4257 (2021).

56 Ak, R., Yilmaz, E., Seyhan, A. U. & Doganay, F. Recurrence of COVID-19 Documented with RT-PCR. *Journal of the College of Physicians and Surgeons--Pakistan : JCPSP* **30**, S26-s28, doi:10.29271/jcpsp.2021.01.S26 (2021).

57 Alshukairi, A. N. *et al.* Re-infection with a different SARS-CoV-2 clade and prolonged viral shedding in a hematopoietic stem cell transplantation patient. *International journal of infectious diseases : IJID : official publication of the International Society for Infectious Diseases* **110**, 267-271, doi:10.1016/j.ijid.2021.07.036 (2021).

58 Alzedam, A., Bengblya, A. M., Zeglam, M. J., Benmassoud, E. T. & Bennji, S. M. A case of COVID-19 re-infection in Libya. *African journal of thoracic and critical care medicine* **27**, doi:10.7196/AJTCCM.2021.v27i2.131 (2021).

59 Atici, S. *et al.* Symptomatic recurrence of SARS-CoV-2 infection in healthcare workers recovered from COVID-19. *Journal of infection in developing countries* **15**, 69-72, doi:10.3855/jidc.14305 (2021).

60 Baiswar, S., Mittal, R., Tiwary, T. & Jinnur, P. Re-Positive SARS-CoV-2 With Respiratory Failure and Cerebrovascular Accident: Is This a Reinfection? *Cureus* **13**, e15825, doi:10.7759/cureus.15825 (2021).

61 Bellesso, M., Bruniera, F. R., Trunkel, A. T. & Nicodemo, I. P. Second COVID-19 infection in a patient with multiple myeloma in Brazil - reinfection or reactivation? *Hematology, transfusion and cell therapy* **43**, 109-111, doi:10.1016/j.htct.2020.12.002 (2021).

62 Chan, P. K. S. *et al.* Serologic Responses in Healthy Adult with SARS-CoV-2 Reinfection, Hong Kong, August 2020. *Emerging infectious diseases* **26**, 3076-3078, doi:10.3201/eid2612.203833 (2020).

63 Díaz, Y. *et al.* SARS-CoV-2 reinfection with a virus harboring mutation in the Spike and the Nucleocapsid proteins in Panama. *International journal of infectious diseases : IJID : official publication of the International Society for Infectious Diseases* **108**, 588-591, doi:10.1016/j.ijid.2021.06.004 (2021).

64 Elzein, F. *et al.* Reinfection, recurrence, or delayed presentation of COVID-19? Case series and review of the literature. *Journal of infection and public health* **14**, 474-477, doi:10.1016/j.jiph.2021.01.002 (2021).

65 Fageeh, H. *et al.* Re-infection of SARS-CoV-2: A case in a young dental healthcare worker. *Journal of infection and public health* **14**, 685-688, doi:10.1016/j.jiph.2021.02.012 (2021).

66 Fakharian, A., Ebrahimibagha, H., Mirenayat, M. S. & Farahmandi, F. COVID-19 Reinfection in a Patient with Hodgkin Lymphoma: a Case Report. *Tanaffos* **20**, 71-74 (2021).

67 Fernandes, A. C. & Figueiredo, R. SARS-CoV-2 reinfection: a case report from Portugal. *Revista da Sociedade Brasileira de Medicina Tropical* **54**, e0002-2021, doi:10.1590/0037-8682-0002-2021 (2021).

68 Ferrante, L. *et al.* The First Case of Immunity Loss and SARS-CoV-2 Reinfection by the Same Virus Lineage in Amazonia. *Journal of racial and ethnic health disparities* **8**, 821-823, doi:10.1007/s40615-021-01084-7 (2021).

69 Garduño-Orbe, B. *et al.* SARS-CoV-2 Reinfection among Healthcare Workers in Mexico: Case Report and Literature Review. *Medicina (Kaunas, Lithuania)* **57**, doi:10.3390/medicina57050442 (2021).

70 Gidari, A. *et al.* Is recurrence possible in coronavirus disease 2019 (COVID-19)? Case series and systematic review of literature. *European journal of clinical microbiology & infectious diseases : official publication of the European Society of Clinical Microbiology* **40**, 1-12, doi:10.1007/s10096-020-04057-6 (2021).

71 Gulati, K., Prendecki, M., Clarke, C., Willicombe, M. & McAdoo, S. COVID-19 Reinfection in a Patient Receiving Immunosuppressive Treatment for Antineutrophil Cytoplasmic Antibody-Associated Vasculitis. *Arthritis & rheumatology (Hoboken, N.J.)* **73**, 1091-1092, doi:10.1002/art.41671 (2021).

72 Habadi, M. I., Balla Abdalla, T. H., Hamza, N. & Al-Gedeei, A. COVID-19 Reinfection. *Cureus* **13**, e12730, doi:10.7759/cureus.12730 (2021).

73 Hanif, M., Haider, M. A., Ali, M. J., Naz, S. & Sundas, F. Reinfection of COVID-19 in Pakistan: A First Case Report. *Cureus* **12**, e11176, doi:10.7759/cureus.11176 (2020).

74 Hayes, B., Stanley, J. & Peppers, B. P. COVID-19 Recurrence Without Seroconversion in a Patient With Mannose-Binding Lectin Deficiency. *Allergy & rhinology (Providence, R.I.)* **12**, 21526567211024140, doi:10.1177/21526567211024140 (2021).

75 Hunsinger, D. H. P., Kutti Sridharan, D. G., Rokkam, D. & Fantry, D. L. E. COVID-19 Reinfection in An Immunosuppressed Patient Without An Antibody Response. *The American journal of the medical sciences* **362**, 103, doi:10.1016/j.amjms.2021.02.003 (2021).

76 Hussein, N. R., Musa, D. H., Saleem, Z. S. M., Naqid, I. A. & Ibrahim, N. Possible COVID-19 reinfection case in Duhok City, Kurdistan: A case report. *Journal of family medicine and primary care* **10**, 2035-2037, doi:10.4103/jfmpc.jfmpc_2396_20 (2021).

77 Ibrahim, M. *et al.* Reinfection versus failure of viral clearance in a COVID-19 patient with hematologic malignancy. *Leukemia research* **101**, 106514, doi:10.1016/j.leukres.2021.106514 (2021).

78 Krishna, V. N., Ahmad, M., Overton, E. T. & Jain, G. Recurrent COVID-19 in Hemodialysis: A Case Report of 2 Possible Reinfections. *Kidney medicine* **3**, 447-450, doi:10.1016/j.xkme.2021.02.004 (2021).

79 Loconsole, D. *et al.* Symptomatic SARS-CoV-2 Reinfection in a Healthy Healthcare Worker in Italy Confirmed by Whole-Genome Sequencing. *Viruses* **13**, doi:10.3390/v13050899 (2021).

80 Mohseni, M., Albus, M., Kaminski, A. & Harrison, M. F. A Case of COVID-19 Re-Infection in a Liver Transplant Patient. *Cureus* **13**, e14916, doi:10.7759/cureus.14916 (2021).

81 Nachmias, V., Fusman, R., Mann, S. & Koren, G. The first case of documented Covid-19 reinfection in Israel. *IDCases* **22**, e00970, doi:10.1016/j.idcr.2020.e00970 (2020).

82 Nonaka, C. K. V. *et al.* Genomic Evidence of SARS-CoV-2 Reinfection Involving E484K Spike Mutation, Brazil. *Emerging infectious diseases* **27**, 1522-1524, doi:10.3201/eid2705.210191 (2021).

83 Novazzi, F. *et al.* SARS-CoV-2 B.1.1.7 reinfection after previous COVID-19 in two immunocompetent Italian patients. *Journal of medical virology* **93**, 5648-5649, doi:10.1002/jmv.27066 (2021).

84 Pow, T., Allen, S., Brailovsky, Y. & Darki, A. Acute submassive pulmonary embolism after SARS-CoV-2 infection: a case report of reinfection or prolonged hypercoagulable state. *European heart journal. Case reports* **5**, ytab103, doi:10.1093/ehjcr/ytab103 (2021).

85 Romano, C. M. *et al.* SARS-CoV-2 reinfection caused by the P.1 lineage in Araraquara city, Sao Paulo State, Brazil. *Revista do Instituto de Medicina Tropical de Sao Paulo* **63**, e36, doi:10.1590/s1678-9946202163036 (2021).

86 Salehi-Vaziri, M. *et al.* Clinical characteristics of SARS-CoV-2 by re-infection vs. reactivation: a case series from Iran. *European journal of clinical microbiology & infectious diseases : official publication of the European Society of Clinical Microbiology* **40**, 1713-1719, doi:10.1007/s10096-021-04221-6 (2021).

87 Santiago-Espinosa, O., Prieto-Torres, M. E. & Cabrera-Gaytán, D. A. Laboratory-conﬁrmed SARS-CoV-2 reinfection in the population treated at social security. *Respiratory medicine case reports* **34**, 101493, doi:10.1016/j.rmcr.2021.101493 (2021).

88 Scarpati, G., Piazza, O., Pagliano, P. & Rizzo, F. COVID-19: a confirmed case of reinfection in a nurse. *BMJ case reports* **14**, doi:10.1136/bcr-2021-244507 (2021).

89 Selvaraj, V., Herman, K. & Dapaah-Afriyie, K. Severe, Symptomatic Reinfection in a Patient with COVID-19. *Rhode Island medical journal (2013)* **103**, 24-26 (2020).

90 Sharma, R. *et al.* A Patient with Asymptomatic SARS-CoV-2 Infection Who Presented 86 Days Later with COVID-19 Pneumonia Possibly Due to Reinfection with SARS-CoV-2. *The American journal of case reports* **21**, e927154, doi:10.12659/ajcr.927154 (2020).

91 Sicsic, I., Jr. *et al.* A case of SARS-CoV-2 reinfection in a patient with obstructive sleep apnea managed with telemedicine. *BMJ case reports* **14**, doi:10.1136/bcr-2020-240496 (2021).

92 Siqueira, J. D. *et al.* Distinguishing SARS-CoV-2 bonafide re-infection from pre-existing minor variant reactivation. *Infection, genetics and evolution : journal of molecular epidemiology and evolutionary genetics in infectious diseases* **90**, 104772, doi:10.1016/j.meegid.2021.104772 (2021).

93 Tang, C. Y. *et al.* Reinfection with two genetically distinct SARS-CoV-2 viruses within 19 days. *Journal of medical virology* **93**, 5700-5703, doi:10.1002/jmv.27154 (2021).

94 Tillett, R. L. *et al.* Genomic evidence for reinfection with SARS-CoV-2: a case study. *The Lancet. Infectious diseases* **21**, 52-58, doi:10.1016/s1473-3099(20)30764-7 (2021).

95 Tomkins-Tinch, C. H. *et al.* SARS-CoV-2 Reinfection in a Liver Transplant Recipient. *Annals of internal medicine* **174**, 1178-1180, doi:10.7326/l21-0108 (2021).

96 Torres, D. A. *et al.* Reinfection of COVID-19 after 3 months with a distinct and more aggressive clinical presentation: Case report. *Journal of medical virology* **93**, 1857-1859, doi:10.1002/jmv.26637 (2021).

97 Tuan, J., Spichler-Moffarah, A. & Ogbuagu, O. A new positive SARS-CoV-2 test months after severe COVID-19 illness: reinfection or intermittent viral shedding? *BMJ case reports* **14**, doi:10.1136/bcr-2020-240531 (2021).

98 Ul-Haq, Z. *et al.* First documented reinfection of SARS-COV-2 in second wave from Pakistan. *Journal of Ayub Medical College, Abbottabad : JAMC* **32(Suppl 1)**, S704-s705 (2020).

99 Vora, T., Vora, P., Vora, F., Sharma, K. & Desai, H. D. Symptomatic reinfection with COVID-19: A first from Western India. *Journal of family medicine and primary care* **10**, 1496-1498, doi:10.4103/jfmpc.jfmpc_2002_20 (2021).

100 West, J., Everden, S. & Nikitas, N. A case of COVID-19 reinfection in the UK. *Clinical medicine (London, England)* **21**, e52-e53, doi:10.7861/clinmed.2020-0912 (2021).

101 Yadav, S. P. *et al.* COVID-19 reinfection in two children with cancer. *Pediatric hematology and oncology* **38**, 403-405, doi:10.1080/08880018.2020.1855276 (2021).

102 Yu, A. L. F. *et al.* SARS-CoV-2 reinfection: report of two cases in Southeast Brazil. *Revista do Instituto de Medicina Tropical de Sao Paulo* **63**, e50, doi:10.1590/s1678-9946202163050 (2021).

103 Zanferrari, C. *et al.* Focal Cerebral Arteriopathy in a Young Adult Following SARS-CoV2 Reinfection. *Journal of stroke and cerebrovascular diseases : the official journal of National Stroke Association* **30**, 105944, doi:10.1016/j.jstrokecerebrovasdis.2021.105944 (2021).

104 Zayet, S. *et al.* Recurrence of COVID-19 after recovery ? A case series in health care workers, France. *Microbes and infection* **23**, 104803, doi:10.1016/j.micinf.2021.104803 (2021).

105 Zhang, E., Lequesne, E., Rohs, A. & Frankle, W. G. Suspected Recurrence of Symptomatic COVID-19: Management During Inpatient Psychiatric Treatment. *Journal of psychiatric practice* **27**, 137-144, doi:10.1097/pra.0000000000000534 (2021).

106 Yao, M. Q. *et al.* Factors associated with a SARS-CoV-2 recurrence after hospital discharge among patients with COVID-19: systematic review and meta-analysis. *Journal of Zhejiang University. Science. B* **21**, 940-947, doi:10.1631/jzus.B2000304 (2020).

107 Piri, S. M., Edalatfar, M., Shool, S., Jalalian, M. N. & Tavakolpour, S. A systematic review on the recurrence of SARS-CoV-2 virus: frequency, risk factors, and possible explanations. *Infectious diseases (London, England)* **53**, 315-324, doi:10.1080/23744235.2020.1871066 (2021).

108 Wang, J., Kaperak, C., Sato, T. & Sakuraba, A. COVID-19 reinfection: a rapid systematic review of case reports and case series. *Journal of investigative medicine : the official publication of the American Federation for Clinical Research* **69**, 1253-1255, doi:10.1136/jim-2021-001853 (2021).

109 Sotoodeh Ghorbani, S. *et al.* Epidemiologic characteristics of cases with reinfection, recurrence, and hospital readmission due to COVID-19: A systematic review and meta-analysis. *Journal of medical virology*, doi:10.1002/jmv.27281 (2021).

110 Quick, J. nCoV-2019 sequencing protocol v3 (LoCost). protocols.io <https://protocols.io/view/ncov-2019-sequencing-protocol-v3-locost-bh42j8ye>. (2020).
